# Correlation of circulating ANGPTL5 levels with obesity, high sensitivity C-reactive protein and oxidized low-density lipoprotein in adolescents

**DOI:** 10.1038/s41598-020-63076-7

**Published:** 2020-04-14

**Authors:** Maha M. Hammad, Mohamed Abu-Farha, Abdullah Al-Taiar, Nada Alam-Eldin, Reem Al-Sabah, Lemia Shaban, Fahd Al-Mulla, Jehad Abubaker, Abdur Rahman

**Affiliations:** 1 Biochemistry and Molecular Biology Department, Dasman Diabetes Institute, Kuwait City, Kuwait; 2 School of Community & Environmental Health, College of Health Sciences, Old Dominion University, Norfolk, VA USA; 3 Department of Community Medicine and Behavioural Sciences, Faculty of Medicine, Kuwait University, Kuwait City, Kuwait; 4 Department of Food Science and Nutrition, College of Life Sciences, Kuwait University, Kuwait City, Kuwait; 5 Genetics and Bioinformatics Department, Dasman Diabetes Institute, Kuwait City, Kuwait

**Keywords:** Dyslipidaemias, Obesity

## Abstract

Angiopoietin-like proteins (ANGPTL) is a family of eight members known to play an important role in metabolic diseases. Of these, ANGPTL5 is suggested to regulate triglyceride metabolism and is increased in obesity and diabetes. However, its role in metabolic diseases in adolescents is not well-studied. In this study, we tested the hypothesis of a positive association between plasma ANGPTL5, and obesity, high sensitivity C-reactive protein (HsCRP) and oxidized low-density lipoprotein (Ox-LDL) in adolescents. Adolescents (N = 431; age 11–14 years) were randomly selected from middle schools in Kuwait. Obesity was classified by the BMI-for-age based on the WHO growth charts. Plasma ANGPTL5, HsCRP, and Ox-LDL were measured using ELISA. The prevalence of overweight and obesity was 20.65% and 33.18%, respectively. Mean (SD) plasma ANGPTL5 levels were significantly higher in obese, compared with overweight and normal-weight adolescents (23.05 (8.79) vs 18.39 (7.08) ng/mL, and 18.26 (6.95) ng/ml, respectively). ANGPTL5 was positively associated with both HsCRP (ρ=0.27, p < 0.001) and Ox-LDL (ρ = 0.24, p < 0.001). In Conclusion, ANGPTL5 levels are elevated in obese adolescents and are associated with cardiovascular disease risk factors, HsCRP and Ox-LDL. The use of ANGPTL5 as a powerful diagnostic and prognostic tool in obesity and metabolic diseases needs to be further evaluated.

## Introduction

Childhood obesity has emerged as a major public health problem worldwide. In Arab states in the Gulf region, extremely high prevalence of childhood obesity has been reported^1^. On a sample of 13, 000 school children each year, Kuwait Nutritional Surveillance has consistently reported that more than 45% of school children aged 5–19 are either overweight or obese^2^. Similarly, a cross-sectional study conducted on 635 public intermediate school children reported that one quarter of the children (25.5%) were overweight and over one third (36.5%) were classified as obese^3^. In-door lifestyle, lack of physical activity and changes in the dietary pattern have been implicated as the underlying causes of childhood obesity in the region. According to the World Health Organization (WHO), the worldwide prevalence of obesity nearly tripled between 1975 and 2016 and is expected to double in the next 25 years. This highlights the importance of dealing with this epidemic. Given the widespread prevalence of obesity and its long-term and short-term complications, one approach to the problem is to focus on the key molecular pathways involved in the development of childhood obesity. This would help identify potential biomarkers that might be used to guide preventive and therapeutic interventions.

Angiopoietin-like protein 5 (ANGPTL5) is a member of the angiopoietin-like proteins (ANGPTLs) family. Eight members have been described so far (ANGPTL1 through ANGPTL8), and have been shown to have various physiological functions in lipid and glucose metabolism, inflammation, hematopoiesis, angiogenesis and cancer^4–7^. They are also differentially expressed in multiple tissues: for example, while ANGPTL3 is exclusively expressed in the liver and kidney, ANGPTL7 is expressed in the eye. Other members such as ANGPTL2 and ANGPTL4 are more ubiquitously expressed. ANGPTL5, on the other hand, was mainly shown to be expressed in adipose tissue, but it was also detected at lower levels in the heart, ovary, testis and skin^5,6,8,9^. ANGPTLs were thought to be orphan ligands until a study showed that ANGPTL1, 2, 5 and 7 can bind to the immune-inhibitory receptor human leukocyte immunoglobulin-like receptor B2^10^.

Our group as well as others investigated the levels of different ANGPTLs in disease conditions in adults and demonstrated significant differences and associations between certain ANGPTL proteins and obesity, insulin resistance, glucose tolerance and inflammation^11–19^. However, fewer studies examined these proteins and their associations with metabolic diseases in childhood and adolescence. Studies in young participants are crucial as their samples are less influenced by disease and lifestyle changes. In addition, some members of the ANGPTL family, particularly ANGPTL5 and ANGPTL7 are still understudied.

Many studies have reported changes in circulating levels of ANGPTL family proteins members in obesity and diabetes, as well as other metabolic diseases. For example, we have shown that levels of ANGPTL8 (also known as betatrophin) were significantly elevated in obese, diabetics and people with metabolic syndrome compared with individuals without these conditions^14–17^. A prospective cohort study investigated the serum levels of ANGPTL8 and ANGPTL3 in Korean Children^20^. The authors demonstrated that circulating ANGPTL8 levels were positively associated with triglycerides (TG), whereas ANGPTL3 levels were associated with fasting insulin and the homeostasis model assessment of insulin resistance (HOMA-IR). However, the study reported no differences in serum levels of ANGPTL8 or ANGPTL3 between normal-weight and overweight children. Previously, we have also shown that ANGPTL8 was positively associated with high sensitivity C-reactive protein (HsCRP), a well-established marker for inflammation and cardiovascular diseases (CVD)^15^. Unlike other ANGPTL proteins, the role of ANGPTL5 in developing obesity and metabolic diseases remains mostly unknown.

In this study, we aimed to compare the circulating level of ANGPTL5 between obese, overweight and normal-weight adolescents, and to investigate the association between ANGPTL5 and well-established CVD risk factors, namely HsCRP and oxidized low-density lipoprotein (Ox-LDL). We hypothesize that, like other ANGPTL proteins, plasma ANGPTL5 levels are positively associated with obesity, HsCRP, and Ox-LDL.

## Materials and Methods

### Study participants

This is a cross-sectional study that was conducted in selected public middle schools from the State of Kuwait as previously described^21,22^. Study participants were adolescent in the age range of 11–14 years.

### Blood collection and biochemical analyses

A sample of 5 mL of venous blood was collected in gel-containing tubes (SST II Advance, BD Vacutainer) from each child and was analyzed for glucose, complete blood count, iron profile, folate, vitamin B_12_, 25-hydroxyvitamin D, calcium and parathyroid hormone. All the blood tests were conducted in a major secondary care hospital where these tests are routinely performed with strict quality control measures.

### ELISA measurements for ANGPTL5, HsCRP and Ox-LDL

Plasma was obtained from the collected blood after centrifugation, aliquoted and then stored at −80 °C. ANGPTL5 concentrations were determined using ELISA kit (Cat. # E4440h; Wuhan EIAAB Science, China) with optimal dilution 1:25, HsCRP concentrations were determined using ELISA kit (Cat. # HK369; Hycult Biotech) with optimal dilution 1:1000. Finally, Ox-LDL concentrations were determined using ELISA kit (Cat. # K 7810; Immundiagnostik AG, Germany) with optimal dilution 1:10. All measurements described above followed the manufacturer’s instructions.

### Anthropometric measurements and other covariates

Standing height and bodyweight of the study participants were measured in a standardized manner, using digital weight and height scale (Detecto, Webb City, MO, USA) with the participants standing erect without shoes and wearing light clothes. Data on socioeconomic status and other covariates were collected from the parents through a self-administered questionnaire, and from the adolescents using face-to-face interview.

### Statistical analysis

BMI-for-age z-scores were calculated using WHO growth charts. Obesity was defined as BMI-for-age ≥ +3 Standard Deviation (SD), while overweight was defined as BMI-for-age > +2 SD and < +3 SD. All measurements are reported as mean (SD) when the data were normally distributed; otherwise, we report median (interquartile range: IQR). Although ANGPTL5 was normally distributed across BMI categories, the homogeneity of variance assumption was not met. Hence, we used Kruskal-Wallis test followed by Dunn’s test as *post hoc* analysis using Bonferroni adjustment. We used the same approach for Ox-LDL and HsCRP measurements, as these were not normally distributed. The association between ANGPTL5 and each Ox-LDL and HsCRP were depicted graphically and Spearman correlation coefficient was calculated. The association between ANGPTL5 and overweight or obesity was evaluated using multinomial logistic regression. First, crude odds ratios were calculated, then we adjusted for other factors that showed association with overweight and obesity at 20% level of significance. However, only self-reported physical condition that limits physical activity was associated with overweight and obesity, while all other covariates were not. Therefore, we adjusted for this factor in addition to age group (10 years, 12 years, 13 years). Separate analyses were conducted while fitting ANGPTL5, first as a continuous variable and then as a categorical variable. Similar analysis was performed with HsCRP and Ox-LDL. We used Wald test to evaluate the statistical significance in these analyses; associations with p < 0.05 were deemed to be significant.

### Ethics statement

The study was approved by The Ethics Committee at Ministry of Health, Kuwait (No: 2015/248), the Ethics Committee of the Health Sciences Centre, Kuwait University (No: DR/EC/2338) and the Ethical Review Committee at Dasman Diabetes Institute (RA2017-026). Written informed consent was obtained from the parents and verbal ascent was obtained from all the study subjects. We certify that the work conducted in this research complies with the ethical standards recommended by the Helsinki Declaration.

## Results

### Description of the study group and obesity in school adolescents

The demographic characteristics of the study group are summarized in Table 1. The mean (SD) age was 12.32 (0.85) years and 197 (45.71%) were males. Of the 431 participants, 193 (44.78%) were normal-weight, 89 (20.65%) were overweight, and 143 (33.18%) were obese. Only 6 participants (1.39%) were underweight and these were included in normal-weight category in the analysis below. There was no significant difference in the prevalence of obesity or overweight between males and females (p = 0.944).

**Table 1:** Socio-demographic characteristics of 431 adolescents enrolled in the study.

Characteristics		
**Age in years, Mean (SD)**	12.32	(0.86)
	**n**	**%**
**Gender**		
Male	197	45.7
**Nationality**		
Kuwaiti	310	71.93
Non-Kuwait	121	28.07
**Father’s Education**^a^		
No formal education up to intermediate school	62	14.69
Secondary (high school)	108	25.59
Diploma	87	20.62
University & above	165	39.10
**Mother’s Education**^b^		
No formal education up to intermediate school	44	10.30
Secondary (high school)	101	23.65
Diploma	90	21.08
University & above	192	44.96
**Father’s Income**^c^ (Kuwaiti Dinars)		
Less than 500	36	8.59
500 to 1000	98	23.39
1001 to 1500	131	31.26
1501 to 2000	61	14.56
More than 2000	52	12.41
Do not wish to tell	41	9.79
**Mother’s Employment Status**^d^		
Housewife	143	33.81
Paid employment	208	49.17
Others	72	17.02

^a^Missing for 9 participants; ^b^Missing for 4 participants; ^c^Missing for 12 participants; ^d^Missing for 8 participants.

### Plasma ANGPTL5 levels are significantly elevated in obese children

**Figure 1:**
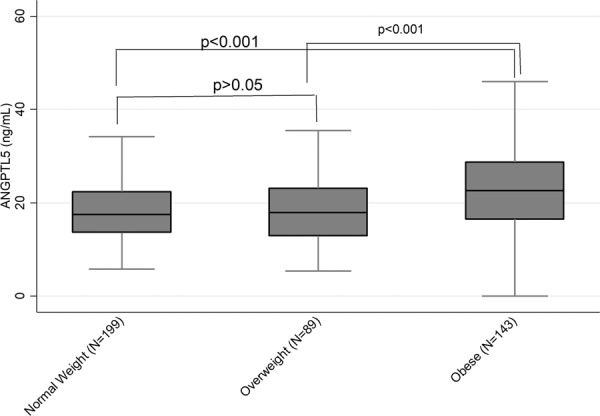
Distribution of Angiopoietin-like protein (ANGPTL5) in normal weight, overweight and obese adolescents.

We found a significant difference in ANGPTL5 levels between obese and normal-weight adolescents, as well as between obese and overweight participants (p < 0.001). The mean (SD) levels of ANGPTL5 were 18.39 (7.08) ng/mL, 18.26 (6.95) ng/mL and 23.05 (8.79) ng/mL in normal-weight, overweight and obese adolescents, respectively **(**Fig. 1**)**. However, levels of ANGPTL5 did not differ between overweight and normal-weight groups (p > 0.05). These results remained unchanged when we excluded underweight adolescents (n = 6) or when we stratified the analysis by gender. Multinomial logistic regression showed that the odds of obesity and overweight was significantly associated with ANGPTL5 in univariable analysis and after adjusting for potential confounders **(**Table 2**)**. This was particularly evident when ANGPTL5 was categorized into tertiles (upper tertile OR [95%CI] = 3.59 [2.32, 5.55]; as compared to the lower tertile [reference] p < 0.001; Table 2).

**Table 2:** Association between overweight/obesity and each ANGPTL5, HsCRP and Ox-LDL in univariable and multivariable multinomial logistic regression.

	Overweight OR [95%CI]	Obesity OR [95%CI]	p-value	Overweight AOR [95%CI]	Obesity AOR [95%CI]	p-value
**ANGPTL5 (ng/mL)**	1.00 [0.96,1.03]	1.08[1.05,1.11]	<0.001	1.00 [0.97,1.03]	1.08[1.06,1.10]	<0.001
**ANGPTL5 (categories)**						
Lower tertile (<16.0 ng/mL)	1[Ref.]	1[Ref.]	<0.001	1[Ref.]	1[Ref.]	0.002
Middle tertile (≥16.0 & <22.6 ng/mL)	0.72[0.35,1.46]	1.12[0.47,2.71]		0.75[0.36,1.54]	1.13[0.48,2.67]	
Upper tertile (≥ 22.6 ng/mL)	1.02[0.53,1.95]	3.59[2.32,5.55]		1.08[0.60,1.98]	3.49[2.25,5.40]	
**HsCRP (µg/mL)**	1.72[1.25,2.38]	2.45[1.77,3.39]	<0.001	1.73[1.25,2.40]	2.45[1.77,3.38]	<0.001
**HsCRP (categories)**						
Lower tertile (<0.28 µg/mL)	1[Ref.]	1[Ref.]	0.004	1[Ref.]	1[Ref.]	0.005
Middle tertile (≥0.28 & <1.54 µg/mL)	5.04[2.47,10.30]	6.99[3.09,15.84]		5.02[2.50,10.11]	7.22[2.79,18.63]	
Upper tertile (≥1.54 µg/mL)	5.23[1.90,14.31]	47.07[15.14,146.35]		5.21[1.94,13.98]	48.49[13.49,168.74]	
**Ox-LDL ng/mL**	1.00[1.00–1.00]	1.00[1.00–1.00]	0.319	1.00[1.00–1.00]	1.00[1.00–1.00]	0.353
**Ox-LDL (categories)**						
Lower tertile (≤138 ng/mL)	1[Ref.]	1[Ref.]	0.237	1[Ref.]	1[Ref.]	0.111
Middle tertile (>138 & ≤406 ng/mL)	1.23 [0.72,2.12]	0.92[0.46,1.81]		1.28 [0.76,2.13]	0.86[0.47,1.58]	
Upper tertile (>406 ng/mL)	0.82[0.46,1.45]	0.70[0.39,1.27]		0.86[0.44,1.69]	0.65[0.36,1.19]	

ANGPTL5: Angiopoietin-like protein 5; AOR: Adjusted odds ratio (adjusted for age, self-reported physical condition that limits physical activity); HsCRP: High sensitivity C-reactive protein; OR: Crude odds ratio; Ox-LDL: Oxidized Low-Density Lipoprotein; Ref.: reference value.

### Obese children have significantly higher levels of HsCRP

**Figure 2:**
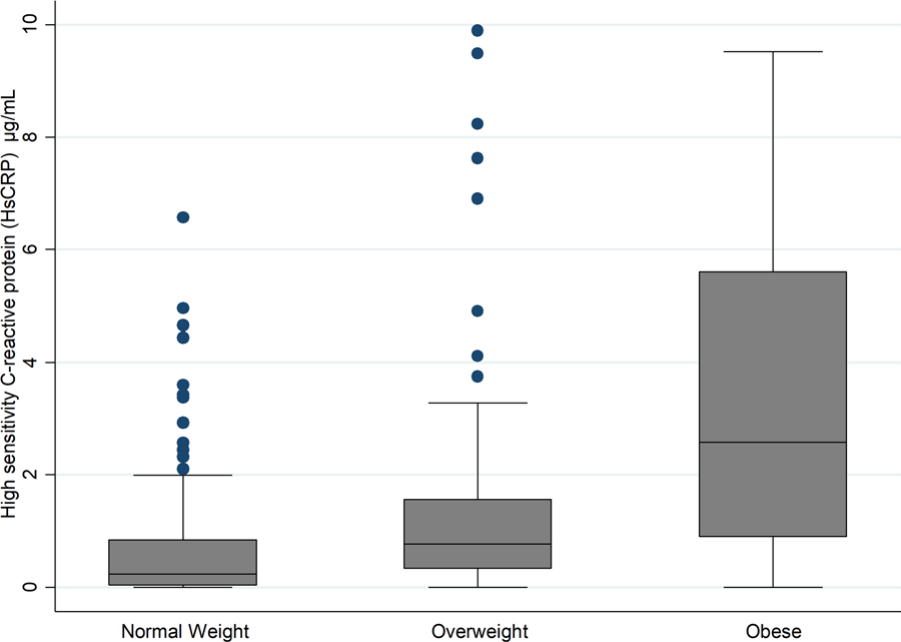
Distribution of high sensitivity C-reactive protein (HsCRP) in normal weight, overweight and obese adolescents.

The median (IQR) levels of HsCRP were 2.57 (0.89–5.60) µg/mL, 0.77 (0.32–1.56) µg/mL, 0.23 (0.04–0.84) µg/mL in obese, overweight and normal-weight participants, respectively **(**Fig. 2**)**. *Post hoc* analysis showed significant differences in levels of HsCRP between each two groups (normal-weight vs overweight, overweight vs obese and obese vs normal-weight [p < 0.01]). These results remained unchanged when we excluded underweight adolescents or stratified the analysis by gender. Multinomial logistic regression showed that the odds of obesity and overweight was significantly associated with HsCRP in both univariable and multivariable analyses whether HsCRP was fitted as continuous or categorical factor **(**Table 2**)**.

### Plasma Ox-LDL levels are not associated with obesity in children

The median (IQR) levels of Ox-LDL were 188.15 (88.16–485.66) ng/mL, 242.80 (100.13–554.92) ng/mL and 255.72 (119.05–614.92) ng/mL, in obese, overweight and normal-weight participants, respectively. There was no significant difference in the levels of Ox-LDL between the different weight groups (p = 0.101). Multinomial logistic regression analysis showed no significant association between Ox-LDL and obesity or overweight, neither in univariable nor in multivariable analysis **(**Table 2**)**.

### ANGPTL5 correlates with inflammatory markers

**Figure 3:**
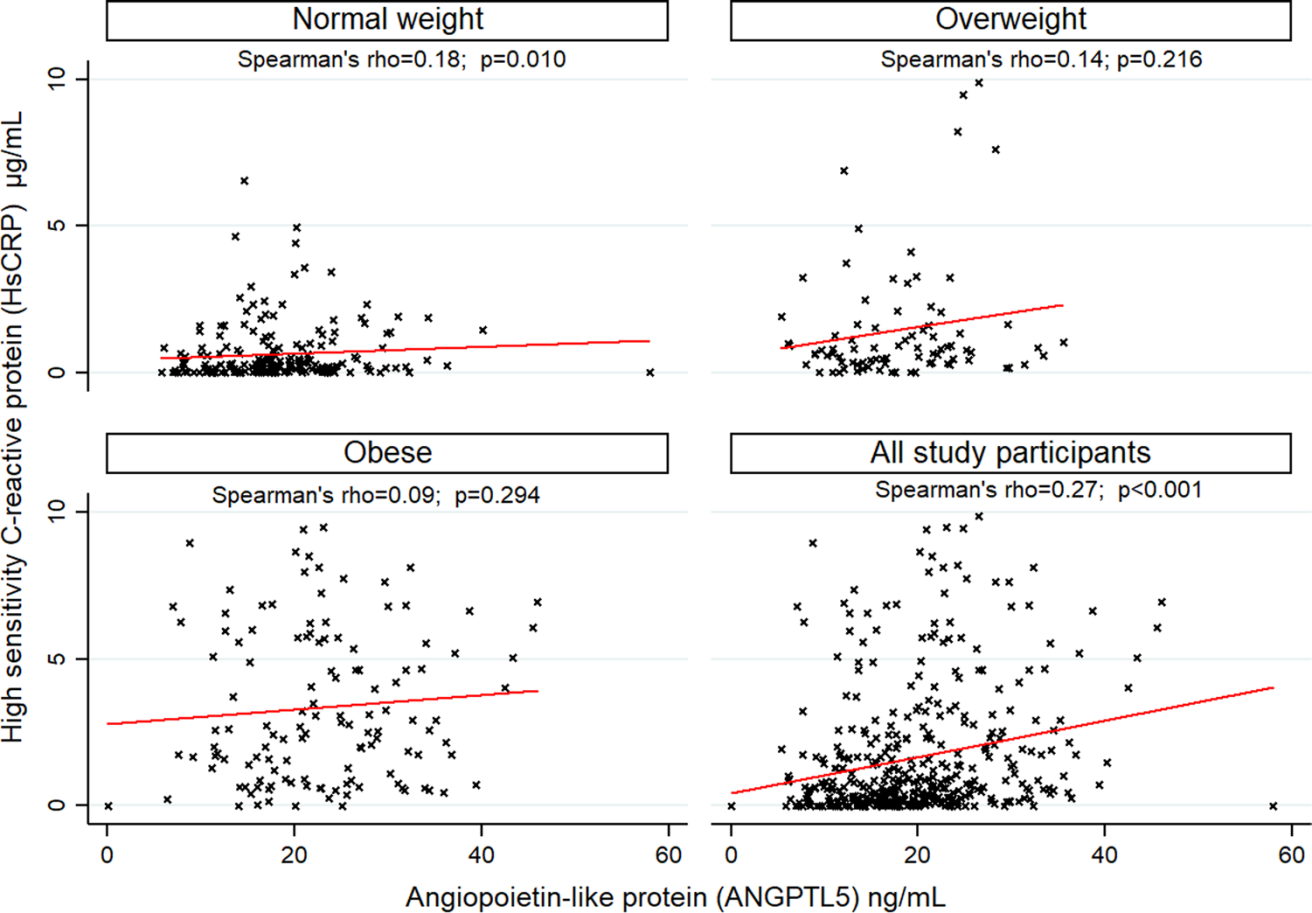
Correlation between Angiopoietin-like protein (ANGPTL5) and high sensitivity C-reactive protein (HsCRP) in 431 adolescents.

**Figure 4:**
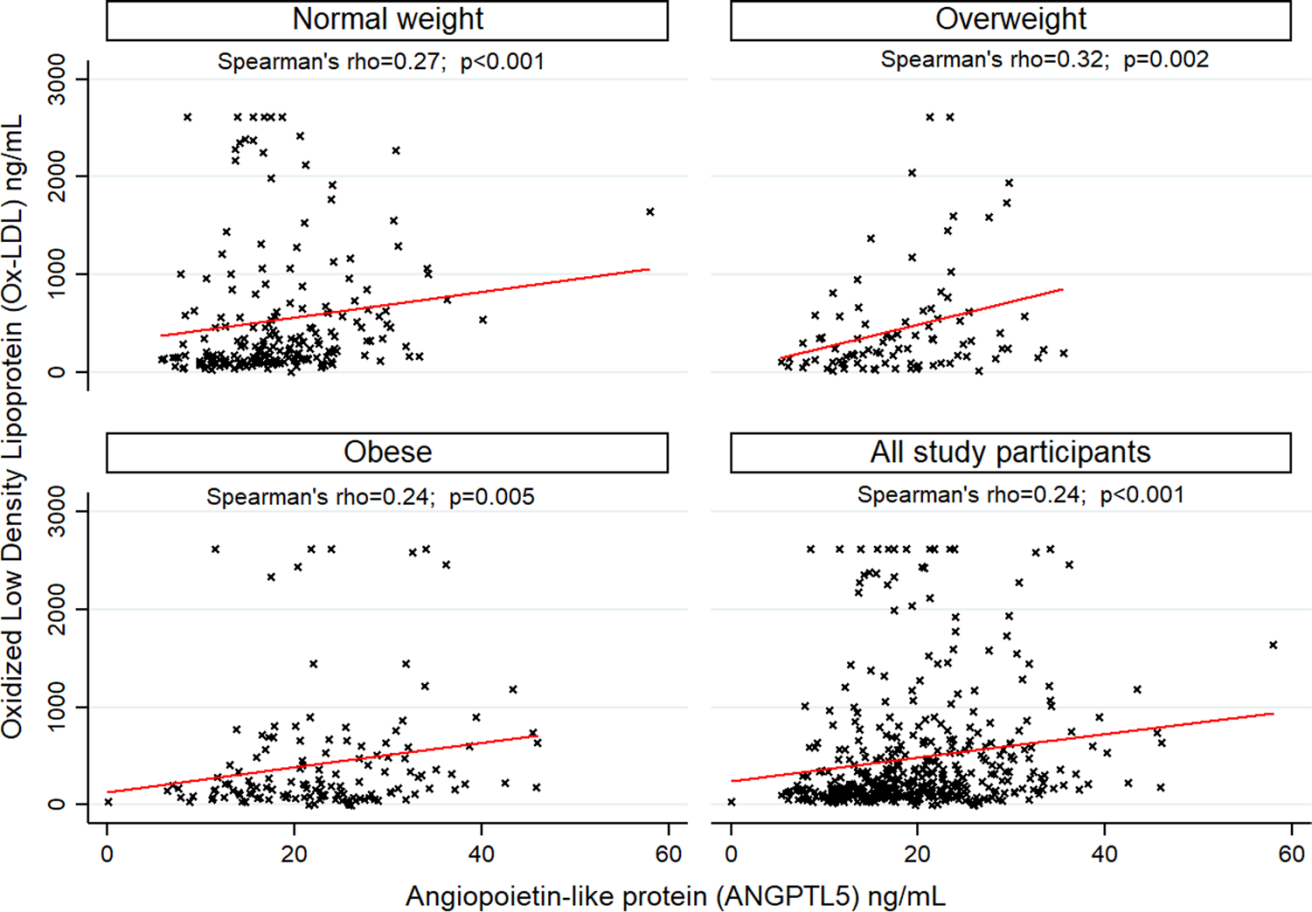
Correlation between Angiopoietin-like protein (ANGPTL5) and Oxidized Low-Density Lipoprotein (Ox-LDL) in 431 adolescents.

We found a positive correlation between ANGPTL5 and HsCRP levels (Spearman’s rho = 0.27, p < 0.001; Fig. 3). This remained evident even after stratification by gender. Circulating ANGPTL5 levels were also positively correlated with Ox-LDL levels (Spearman’s rho = 0.24, p < 0.001) and this correlation was preserved when the population was stratified into normal-weight (Spearman’s rho = 0.27, p < 0.001), overweight (Spearman’s rho = 0.32, p = 0.002), or obese (Spearman’s rho = 0.24, p = 0.005; Fig. 4).

## Discussion

Childhood obesity presents a major public health challenge due to its main role in the development of cardiovascular diseases and diabetes. One of the main pathways that are dysregulated in cardiovascular diseases and diabetes are the ANGPTL proteins. They have been shown to impact metabolic pathways including lipid and glucose metabolism as well as inflammation. Elevated levels of ANGPTL proteins have been linked to obesity, diabetes and metabolic syndrome^11,13,16,17,19^. We recently demonstrated an increase in circulating plasma ANGPTL5 levels in obese vs non-obese adults as well as in adults with and without diabetes^23^. In the current study, we investigated the role of ANGPTL5, a less studied member of the ANGPTL protein family, in childhood obesity and its association with the metabolic markers HsCRP and Ox-LDL.

Our data showed a significant increase in plasma ANGPTL5 levels in obese children compared with normal-weight and overweight children. This finding was further confirmed by multinomial logistic regression analysis, which showed that adolescents with higher levels of plasma ANGPTL5 were 3.5 times more likely to be obese after adjusting for multiple potential confounders. The lack of significant difference in the plasma ANGPTL5 levels between normal-weight and overweight children may indicate that ANGPTL5 is a late obesity marker, and therefore, is not present in higher levels in overweight children. Our findings contrast with the few studies that investigated other ANGPTLs family members, namely ANGPTL3 and ANGPTL8 (betatrophin), and reported no differences between normal-weight and obese children^20,24,25^. This suggests that ANGPTL5 could be a specific and a more sensitive marker for childhood as well as adult obesity than ANGPTL3 and ANGPTL8. On the other hand, this apparent difference could result from the different design of the previous studies, in which overweight and obese participants were placed in the same group, and levels of ANGPTLs levels were compared with normal-weight children. In our study, the stratification into three categories (normal-weight, overweight and obese) allowed us to observe significantly lower levels of ANGPTL5 in overweight subjects compared with obese participants. Therefore, any difference in ANGPTL levels between normal-weight and overweight/obese children in these studies could have been masked by the overweight group. Another striking difference between previous studies and ours is the ethnicity of the study population: while our study subjects were Arabs, previous studies were conducted on Asian children. Further studies with larger populations are needed to confirm the role of ethnicity in the association between ANGPTL5 and obesity.

Obesity is characterized as a low-grade inflammation condition in which various inflammatory markers are increased^26–28^. HsCRP is a powerful marker for detecting low inflammatory processes and is involved in many diseases. It is also regularly used for the diagnosis and prognosis of systemic inflammation^29^. HsCRP is produced in the liver in response to inflammatory signals and cytokines. Elevated CRP levels have been associated with cardiovascular mortality in adults^30^, as well as an increased risk of diabetes and insulin resistance in adults, children and adolescents^31,32^. Parallel to the increased plasma ANGPTL5, plasma HsCRP levels were significantly higher in obese participants compared with normal-weight and overweight subjects. Multinomial logistic regression analysis showed that children with higher levels of HsCRP were 1.7 and 2.4 times more likely to be overweight and obese, respectively.

The strong positive association between ANGPTL5 and obesity on one hand, and between HsCRP and obesity on the other hand, together with the significant positive correlation between ANGPTL5 and HsCRP, suggest that both ANGPTL5 and HsCRP are part of a common pathway involved in the development of obesity. The increase in circulating HsCRP levels is thought to be attributed to the infiltration of the expanded adipose tissue by macrophages, which are responsible for the generation of inflammatory signals and the production of cytokines such as IL-6. Increased production of inflammatory cytokines subsequently can trigger the production of HsCRP by hepatocytes^33^.

Previous studies have suggested that higher levels of ANGPTL proteins are associated with an increased risk of CVD due to their role in regulating key metabolic pathways, such as triglyceride (TG) metabolism. It is possible that the increased ANGPTL5 can impact TG metabolism. ANGPTL5 has been previously shown to inhibit TG lipolysis alongside ANGPTL3, 4 and 8 through inhibiting the activity of lipoprotein lipase (LPL)^4,5,34–38^. Studies on ANGPTL8 and ANGPTL3 knockout mouse models demonstrated that these ANGPTLs play a key role in the metabolic transition between fasting and feeding states^39,40^. The mechanism of action of ANGPTL8 in the fasted and fed state and its interplay with ANGPTL3 and 4 was recently reviewed^41^. Data from several loss-of-function studies showed that alleles in ANGPTL3, 4 and 5 are associated with lower plasma levels of TG. In addition, 1% of the participants in the Dallas Heart Study and 4% of those with plasma TG in the lowest quartile had a rare loss-of-function mutation in one of the three genes^36^. Furthermore, sequencing ANGPTL5 revealed a significant excess of non-synonymous variants in the lowest quartile of TG levels compared with the highest quartile. Overall, 3 out of the 7 missense mutations in ANGPTL5 associated with a low plasma TG level significantly reduced the secretion of ANGPTL5 protein while the wild-type and I233V ANGPTL5 (the allele containing the missense mutation found in the high TG group) did not interfere with protein secretion. However, in this study, the low level of expression of ANGPTL5 compared to ANGPTL3 and 4, did not allow for studying the effect of ANGPTL5 mutations on LPL-mediated hydrolysis of TG^36^. It has also been challenging to further explore the functional role of ANGPTL5 in regulating TG metabolism because of the lack of a mouse ortholog. Taken together, the available data suggest a common role for some ANGPTLs, including ANGPTL5, in the metabolism of TG. It also seems that these proteins are not functionally redundant since the sequence variations in the different genes contribute independently to plasma TG levels. Another potential mechanism through which ANGPTL may increase the risk of CVD is the triggering of inflammation and the oxidation of LDL. Inflammation is the initiating trigger for CVD. The two well-established CVD risk factor are HsCRP and Ox-LDL. The strong positive correlation of ANGPTL5 with both known biomarkers for CVD clearly suggests a role for ANGPTL5 in the pathogenesis of CVD.

Finally, it is important to acknowledge that several approaches are being developed to target ANGPTLs because of their role in TG metabolism. One example is controlling ANGPTL levels by cooling. The rationale behind this approach comes from the many reported beneficial effects of activating brown adipose tissue (BAT) stimulated by cold exposure. The first ANGPTL that was shown to be affected by cold exposure was ANGPTL4^42,43^. Cold exposure increased ANGPTL4 expression in adipose tissue of mice and increased serum ANGPTL4 levels in healthy men. A more recent paper reported that short-term cooling can elevate the levels of ANGPTL3, 4 and 8 in the plasma of young healthy lean men; however, in middle-aged men who were overweight and had prediabetes, only the levels of ANGPTL4 were increased with no effect on the levels of ANGPTL3 and 8^44^. Another emerging approach for targeting ANGPTL proteins is the use of monoclonal antibodies and antisense oligonucleotides. Despite being at early stages, and still limited to ANGPTL3, 4 and 8, this strategy is expected to have significant therapeutic effects on metabolic diseases^45^. For example, a recent study showed that the ANGPTL8 antisense oligonucleotide can improve adipose lipid metabolism and prevent diet-induced non-alcoholic fatty liver disease and hepatic insulin resistance in rodents^46^.

Our study has several strengths. First, to the best of our knowledge, this is the first study investigating the association between ANGPTL5 and obesity and biomarkers for CVD in adolescents. Previous studies are all based on adult populations, which may not be relevant to childhood and adolescent obesity. Second, our study is based on a reasonably large sample size, representative of the Kuwaiti adolescent population. Third, we employed several statistical approaches to robustly show the association between ANGPTL5 and obesity. Fourth, the data were adjusted for many potential confounding factors. There are, however, several limitations in this study as well. First, we were unable to obtain data on insulin resistance and lipid profile of the participants. Given that the original study was designed to analyze the association between vitamin D levels and cognitive function, it was neither necessary nor logistically possible to obtain fasting blood samples required for insulin resistance and lipid profile analyses. As such we could not evaluate the association of ANGPTL5 with plasma TG levels in this population. Second, the cross-sectional design of the study did not allow us to establish causality and requires further investigation to establish the role of ANGPTL5 in the development of obesity and the nature of its association with Ox-LDL and HsCRP. Finally, the antibody in the ELISA kit that we used for measuring ANGPTL5 recognizes the full proteins and is not specific for a particular fragment of the protein. Various fragments of the ANGPTL5 protein could display different functions as displayed by ANGPTL3 for example. Whether ANGPTL5 displays such fragment-specific effects remains to be investigated.

In conclusion, we have demonstrated that ANGPTL5 and HsCRP were increased in obese adolescents in our population. This increase in ANGPTL5 was associated with an increased obesity risk. ANGPTL5 showed strong positive correlation with the well-known CVD risk factors HsCRP and Ox-LDL. These findings highlight the importance of ANGPTL5 in adolescent obesity and metabolic diseases and highlights the need for further studies to characterize the potential use of ANGPTL5 as a powerful diagnostic and prognostic tool for obesity and metabolic diseases.

## Data Availability

The datasets used and/or analyzed during the current study are available from the corresponding author on reasonable request.
